# Deletion of CD38 Suppresses Glial Activation and Neuroinflammation in a Mouse Model of Demyelination

**DOI:** 10.3389/fncel.2019.00258

**Published:** 2019-06-06

**Authors:** Jureepon Roboon, Tsuyoshi Hattori, Hiroshi Ishii, Mika Takarada-Iemata, Thuong Manh Le, Yoshitake Shiraishi, Noriyuki Ozaki, Yasuhiko Yamamoto, Akira Sugawara, Hiroshi Okamoto, Haruhiro Higashida, Yasuko Kitao, Osamu Hori

**Affiliations:** ^1^Department of Neuroanatomy, Graduate School of Medical Sciences, Kanazawa University, Kanazawa, Japan; ^2^Department of Functional Anatomy, Graduate School of Medical Sciences, Kanazawa University, Kanazawa, Japan; ^3^Department of Biochemistry and Molecular Vascular Biology, Graduate School of Medical Sciences, Kanazawa University, Kanazawa, Japan; ^4^Department of Molecular Endocrinology, Tohoku University Graduate School of Medicine, Sendai, Japan; ^5^Department of Biochemistry, Tohoku University Graduate School of Medicine, Sendai, Japan; ^6^Research Center for Child Mental Development, Kanazawa University, Kanazawa, Japan

**Keywords:** demyelination, gliosis, cuprizone, neuroinflammation, NAD^+^

## Abstract

CD38 is an enzyme that catalyzes the synthesis of cyclic adenosine diphosphate-ribose from nicotinamide adenine dinucleotide (NAD^+^). We recently reported that this molecule regulates the maturation and differentiation of glial cells such as astrocytes and oligodendrocytes (OLs) in the developing brain. To analyze its role in the demyelinating situation, we employed cuprizone (CPZ)-induced demyelination model in mice, which is characterized by oligodendrocyte-specific apoptosis, followed by the strong glial activation, demyelination, and repopulation of OLs. By using this model, we found that CD38 was upregulated in both astrocytes and microglia after CPZ administration. Experiments using wild-type and CD38 knockout (KO) mice, together with those using cultured glial cells, revealed that CD38 deficiency did not affect the initial decrease of the number of OLs, while it attenuated CPZ-induced demyelination, and neurodegeneration. Importantly, the clearance of the degraded myelin and oligodendrocyte repopulation were also reduced in CD38 KO mice. Further experiments revealed that these observations were associated with reduced levels of glial activation and inflammatory responses including phagocytosis, most likely through the enhanced level of NAD^+^ in CD38-deleted condition. Our results suggest that CD38 and NAD^+^ in the glial cells play a critical role in the demyelination and subsequent oligodendrocyte remodeling through the modulation of glial activity and neuroinflammation.

## Introduction

Multiple sclerosis (MS) is a chronic inflammatory, demyelinating, and degenerative disease of the central nervous system (CNS), and has been one of the leading causes of neurological disability among young adults. Although MS is generally considered an autoimmune disorder (Compston and Coles, 2008), evidence suggests that the activation of glial cells is also a prominent feature of the demyelinating lesions (Cao and He, 2013; Bond et al., 2014), and that progressive MS is associated with chronic activation of glial cells in the CNS (Lassmann et al., 2012).

Cuprizone (CPZ)-induced experimental demyelination is a suitable rodent model to study the mechanisms leading to demyelination and subsequent remyelination in the CNS without a significant autoimmune lymphocytic response (Remington et al., 2007). In this model, the first pathological event is the selective apoptotic death of mature oligodendrocytes (OLs) in particular brain regions such as the corpus callosum (CC), which results in primary demyelination (Blakemore, 1972). However, the pathology of CPZ-induced demyelination also includes prominent astrocytic reactions and microglial invasion (Praet et al., 2014). In this model, astrocytes were suggested to exert an immune response through the expression of cytokines and recruitment of microglia to demyelinating lesions (Williams et al., 2007; Gudi et al., 2014). Recent studies demonstrated that ablation of astrocytes or astrocyte-targeted production of interleukin-6 led to a reduction in the activation and invasion of microglia into demyelinating lesions, which resulted in delayed demyelination and subsequently delayed oligodendrocyte precursor cell (OPC) proliferation, and remyelination (Skripuletz et al., 2013; Petkovic et al., 2016). Regarding microglial activation, some *in vitro* data suggest that CPZ-induced OL apoptosis requires the presence of microglia-derived proinflammatory mediators such as TNFα and inducible nitric oxide synthase (iNOS) (Pasquini et al., 2007; Raposo et al., 2013).

CD38 is an enzyme that catalyzes the synthesis of cyclic adenosine diphosphate-ribose (cADPR) from nicotinamide adenine dinucleotide (NAD^+^) (Lee, 2001; Malavasi et al., 2008). CD38 has been shown to promote the secretion of oxytocin from hypothalamic neurons and insulin from pancreatic beta cells (Takasawa et al., 1993; Jin et al., 2007). In the brain, however, CD38 expression has been observed not only in oxytocin neurons, but also in glial cells, including astrocytes and microglia (Yamada et al., 1997; Akimoto et al., 2013). We have demonstrated that astrocytic CD38 regulates maturation of astrocytes and differentiation of OPCs into OLs by consuming NAD^+^ in the brain under physiological conditions (Hattori et al., 2017). In pathological conditions, CD38 has been reported to be involved in the activation of microglia and astrocytes in mouse models of glioma and traumatic brain injury, and human HIV-infected brains (Kou et al., 2009; Levy et al., 2009, 2012). However, the role of CD38 in demyelination is still unclear, although its involvement in the modulation of T-cell activation was reported in the experimental autoimmune encephalomyelitis (EAE), another widely used animal model of MS (Herrmann et al., 2016).

In this study, we investigated the role of CD38 in CPZ-induced demyelination model. We found that CD38 was upregulated in both astrocytes and microglia in the demyelinating area after CPZ administration. CD38 deficiency attenuated glial activation and demyelination, most likely through the enhanced level of NAD^+^, while it suppressed myelin clearance, and OL repopulation. These observations suggest critical roles of CD38 and NAD^+^ in the glial cells in the processes of demyelination and neuroinflammation.

## Materials and Methods

### Chemicals

The chemicals used in this study are as follows: bis-cyclohexanone oxaldihydrazone (CPZ) (C9012, Sigma-Aldrich, St. Louis, MO, United States), LPS (20389-04, Nacalai Tesque, Kyoto, Japan), β-NAD^+^ (24334-97, Nacalai Tesque), 8-bromo-cADPR (8-Br-cADPR) (151898-26-9, Santa Cruz Biotechnology), 3-(4,5-dimethyl-2-thiazolyl)-2,5-diphenyltetrazolium bromide (MTT) (29893-1, Nacalai Tesque), and phenazine methosulfate (PMS) (26712-51, Nacalai Tesque).

### Animals

Wild-type (WT) and CD38 knockout (KO) male ICR mice were used for the experiments (body weight; 30–35 g). CD38 KO mice were generated as described previously, and backcrossed for more than eight times (Kato et al., 1999). All mice were housed at mouse cage (345 mm × 168 mm × 140 mm) in a temperature-controlled room (24°C) with a 12 :12 light-dark cycle. The animals were killed at various time points as described in Section “Results.” All animal experiments were performed in accordance with the guidelines and approved by the Animal Care and Use Committee of Kanazawa University (AP-143305).

### Cuprizone Administration

To induce demyelination in 10-week-old ICR male mice, they were administered a diet containing 0.4% CPZ mixed into standard rodent chow ground into a fine powder, as previously described (Song et al., 2007). The powder was served in small ceramic bowls placed into the cages, which were cleaned twice weekly. The mice were fed CPZ for 1, 2, 3, or 5 weeks. Control mice were fed standard rodent chow.

### Quantitative Real-Time Polymerase Chain Reaction

RNA was extracted from brain tissue or cultured cells using RNeasy^®^ Mini Kit (74106, Qiagen, Hilden, Germany). Total RNA was reverse-transcribed using the High-Capacity cDNA Reverse Transcription Kit (4368814, Applied Biosystems, Warrington, United Kingdom) and analyzed by quantitative real-time polymerase chain reaction (RT-qPCR). RT-qPCR was performed as previously described (Hattori et al., 2010). Individual cDNA sequences were amplified using the Thunderbird^TM^ SYBR qPCR^®^ Mix (QPS-201, Toyobo Co., Ltd.) using specific primers. The comparative Ct method was used for data analyses in MxPro 4.10 (Agilent Technologies). Specific ratio comparisons (gene of interest/*Gapdh*) were used to evaluate differences in transcript expression between groups. The primer sequences are listed in Supplementary Table 1.

### Western Blot Analyses

Samples from brains or cultured cells were homogenized in a buffer containing 1% NP-40, 0.1% sodium dodecyl sulfate (SDS), and 0.2% deoxycholate. Denatured lysates were electrophoretically separated using SDS-polyacrylamide gel electrophoresis, and proteins were transferred onto polyvinylidene fluoride membranes. The membranes were then blocked in 5% skimmed milk for 30 min, incubated with anti-CD38 (AF4947, R&D systems, MN, United States, 1:500), anti-glial fibrillary acidic protein (GFAP) (G9269, Sigma, MO, United States, 1:5,000), anti-ionized calcium binding adaptor molecule 1 (Iba1) (019-19741, Wako, Osaka, Japan, 1:500), or anti-myelin basic protein (MBP) (MAB396, Merck Millipore, Darmstadt, Germany, 1:1,000) antibodies for 12 h at 4°C. The primary and secondary antibodies (for details see Supplementary Table 2) were used according the manufactures’ instructions. The membranes were then incubated with anti-rabbit (SC-2004, 1:5,000), anti-mouse (SC-516102, 1:5,000), anti-goat (SC-2354, 1:1,000) or anti-rat (NA9350, Amersham Pharmacia biotech, 1:1000), horseradish peroxidase-linked immunoglobulin G (Cell Signaling Technology, Tokyo, Japan) for 2 h at room temperature. Immunoreactivity was detected using an enhanced chemiluminescence system (GE Healthcare Bio-Sciences, PA, United States). Densitometric quantification was performed using ImageJ software (https://imagej.nih.gov/ij/).

### Immunohistochemistry

After perfusion with 4% paraformaldehyde (PFA), brains were removed from mice and subjected to post-fixation in 4% PFA, followed by dehydration in 30% sucrose. Twenty micrometer-thick sections from bregma +1.2 mm to bregma -1.4 mm were obtained using a cryostat (CM1950, Leica, Nussloch, Germany). To evaluate demyelination, endogenous peroxidase activity was blocked with 0.3% H_2_O_2_ in 70% methanol for 30 min. After blocking with normal goat serum, the sections were incubated with anti-MBP (MAB396, Merck Millipore, 1:100), anti-degraded MBP (dMBP) (AB5864, Merck Millipre, 1:2000), or anti-Amyloid Precursor Protein (APP) (MAB348, Millipore, 1:200) antibodies for overnight at 4°C. After washing in phosphate-buffered saline (PBS), the sections were incubated for 30 min at room temperature with the secondary antibody (MP-7444, MP7402, MP7401, ImmPRESS reagent, Vector Laboratories, Peterborough, United Kingdom), followed by further incubation in a peroxidase substrate solution [SK-4105, ImmPACT 3′-diaminobenzidine (DAB), Vector Laboratories). Demyelination areas in the CC were analyzed by light microscopy at 20× magnification (BZ-X710, Keyence) and quantified using ImageJ software. To determine the percentage of demyelination, the demyelinating area was divided by the total area.

For the detection of GFAP, Iba1, SMI-32, Platelet-derived growth factor receptor alpha (PDGFRα), adenomatous polyposis coli (APC), the sections were processed for immunostaining with antibodies against GFAP (1:2000), Iba1 (1:500), SMI-32 (801701, Biolegend, CA, United States 1:500), PDGFRα (sc-338, Santa Cruz Biotechnology, 1:500) and APC (OP80, Calbiochem, CA, United States, 1:100). Alexa488- or Cy3-conjugated secondary antibodies were used to visualize immunolabeling. Immunofluorescent staining of primary cultures was performed as previously described (Hattori et al., 2007). Imaging was performed on a laser scanning confocal microscope (Eclipse TE2000U, Nikon, Tokyo, Japan) using Nikon EZ-C1 software or a fluorescence microscope (BZ-X710). The numbers of APC-, GFAP-, and Iba1-positive cells with identified nuclei [4′,6-diamidino-2-phenylindole (DAPI)-stained] in the medial part of the CC from 2 sections per mouse were determined at a magnification of 20× (BZ-X710). The results are presented as the number of cells per mm^2^.

### *In situ* Hybridization-Immunohistochemistry

*In situ* hybridization was performed as previously described (Hattori et al., 2014). cDNA fragments of mouse CD38 were amplified by reverse transcription-PCR using the sense/antisense primer set of 5′-ATGCAGGGCGGGGGTCCCCGG- 3′/5′ -TCAGGCCTCGGTTTCCTGAG- 3′, and used as templates for probe synthesis. In brief, brains were removed from mice after perfusion with PBS and immediately placed at -80°C. Serial 14 μm-thick coronal sections were obtained using a cryostat and hybridized with digoxigenin-labeled *Cd38* RNA probes. After development and thorough washing, the brain sections were subjected to immunohistochemistry using primary antibodies such as polyclonal rabbit anti-GFAP (1:2,000), polyclonal rabbit anti-Iba1 (1:200), and mouse anti-APC (1:100). The sections were incubated with primary antibodies overnight at 4°C, and, after washing, were incubated with the secondary antibody (ImmPRESS reagent, Vector Laboratories) for 30 min at room temperature. After washing with PBS, the sections were incubated in a peroxidase substrate solution (ImmPACT DAB, Vector Laboratories). The numbers of GFAP- and Iba1-positive cells with CD38 mRNA expression in two sections per mouse were determined using a light microscope at a magnification of 40× (BZ-X710). Data are presented as the number of cells per mm^2^.

### Electron Microscopy

Mice were perfused with heparinized ringer solution followed by a fixative consisting of 2% paraformaldehyde and 2.5% glutaraldehyde in 30 mM HEPES-NaOH buffer (pH 7.4). The CC was cut from brains removed 2 h after perfusion and then fixed with the same fixative by immersion at 4circC overnight. After washing with 30 mM HEPES-NaOH buffer (pH 7.4), the tissue blocks were post-fixed with 1% OsO4 for 2 h, dehydrated in increasing concentrations of ethanol, and embedded in Quetol 812 resin (NISSIN EM Co., Tokyo, Japan) at 65°C for 24 h. Sagittal-Vertical ultrathin sections were cut at about 80 nm thickness using an ultramicrotome (LKB-8800, LKB produkter, Bromma, Sweden) and collected on nickel grids (NISSIN EM Co.). The sections were stained with uranyl acetate and lead citrate, and analyzed in a transmission electron microscope (EM; JOEL, Tokyo, Japan) using an accelerating voltage of 80 kV. The photographs were analyzed using Image J Software to calculate the g-ratio. The g-ratio was calculated as previously described (Goebbels et al., 2010). To study the demyelinated axons of the CC, serial 1 μm-thick semi-thin sections were cut with a glass knife, stained with 1% toluidine blue, and examined by light microscopy at a magnification of 60× (BZ-X710). The number of myelinated axons was calculated as previously described (Fan et al., 2017). Eight sections, with an area of 650 μm^2^ per section, were evaluated from 4 animals for each group. 100 axons from 2 mice in each group were analyzed to determine the g-ratio.

### Analysis of NAD Levels in the CC

Nicotinamide adenine dinucleotide (NAD^+^ + NADH) levels were determined as previously described (Shibata and Murata, 1986). In brief, the CC was harvested from WT and CD38 KO mice after administration of CPZ for 5 weeks. The tissue was weighed and homogenized in an extraction buffer or 50 mM potassium phosphate buffer (pH 6.0) containing 100 mM nicotinamide using hand-held homogenizers. The samples were centrifuged at 8000 ×*g* for 3 min following incubation at 90°C for 1.5 min. The supernatant (50 μl) was incubated at 37°C for 10 min in 32.5 mM glycylglycine-NaOH buffer, 50 mM nicotinamide, 0.25 M EtOH, 0.083 mg/ml MTT, and 0.27 mg/ml PMS (pH 7.4). After the addition of alcohol dehydrogenase (12.5 IU/ml), the increase in absorbance at 570 nm was monitored using a Multiskan GO Microplate Spectrophotometer (Thermo Fischer Scientific, MA, United States). The values were normalized to milligrams of tissue or protein.

### Glial Cell Cultures

Cerebral cortices from WT and CD38 KO neonatal mice (P1 to P3) were harvested and digested at 37°C by Dispase II (383-02281, 2 mg/mL, Wako). The Cells were plated in 75-cm^2^ culture flasks (Corning) in Dulbecco’s Modified Eagle Medium (DMEM) supplemented with 10% fetal bovine serum (FBS) (172012, Sigma-Aldrich) and penicillin and streptomycin (26239-42, 32204-92, Nacalai Tesque). The cells were collected after 14 days of cultivation. They were then incubated with CD11b (microglia) MicroBeads (130-093-634, microbeads conjugated to monoclonal anti-human/mouse CD11b antibody, Miltenyi Biotec, Bergisch Gladbach, Germany) for 15 min at 4°C. The cells were then washed in separation buffer and centrifuged at 300 ×*g* for 10 min. They were resuspended in the same buffer and applied to a magnetic column fitted into a QuadroMACS^TM^ cell separator (Miltenyi Biotec). The cells were separated into CD11b-positive or CD11b-negative fractions and plated onto poly-L-lysine-coated plastic culture dishes. The CD11b-positive fraction, which contained microglia (>97% of the cells were Iba1 positive), was used for the experiments 24 h after plating. The CD11b-negative fraction, which contained astrocytes (>92% of the cells were GFAP positive), was plated and used for experiments after reaching confluence.

### Small Interference RNA

Silencing of CD38 was performed by transfecting the cells with small interference RNAs (siRNAs), as previously described (Hattori et al., 2017). The targeted sequence of the mouse CD38 was as follows: 5′ -GGACCCAAATAAGGTTCA- 3′. We used Stealth RNAi^TM^ siRNA negative control Med GC from Thermo Fisher Scientific as a control siRNA (12935-300). Microglia were transfected with siRNA 1 day after plating, while astrocytes were transfected with siRNA 4 and 6 days after plating. Two days later, the cells were treated with LPS (100 ng/ml) for 6 h, and total RNA was isolated for qRT-PCR.

### Phagocytosis Assays

The microglia phagocytosis assay was performed using myelin debris with or without LPS at the concentration of 100 ng/ml, as previously described (Clarner et al., 2015). In brief, the CC was harvested under sterile conditions from adult ICR mice, and the cerebral cortices were carefully removed. The samples were homogenized in PBS using an ultrasonic converter and centrifuged at 1,000 ×*g* for 10 min. The supernatant was used as myelin debris. Myelin debris at the concentration of 15 μg/ml was used to assess the phagocytic activity of cells. After 24 h of LPS stimulation, immunocytochemistry was performed using anti-Iba1 (1:200) and anti-MBP (1:200) antibodies overnight at 4°C. Five independent microglia cultures were evaluated using a light microscope at a magnification of 20× (BZ-X710, Keyence). Phagocytic activity is presented as the percentage of myelin-containing microglia in the total number of microglia.

### Statistical Analysis

The experimental results are expressed as mean ± standard error of the mean (SEM), with the number of experiments indicated by (n). No statistical evaluations were performed to predetermine sample sizes, but our sample sizes are similar to those generally used in the field. One-way ANOVA followed by Tukey-kramer test or two-way ANOVA followed by Scheffe’s *F* test was used for the statistical analysis. *P* values <0.05 were considered statistically significant.

## Results

### CD38 Expression Was Increased During CPZ Administration

**FIGURE 1:**
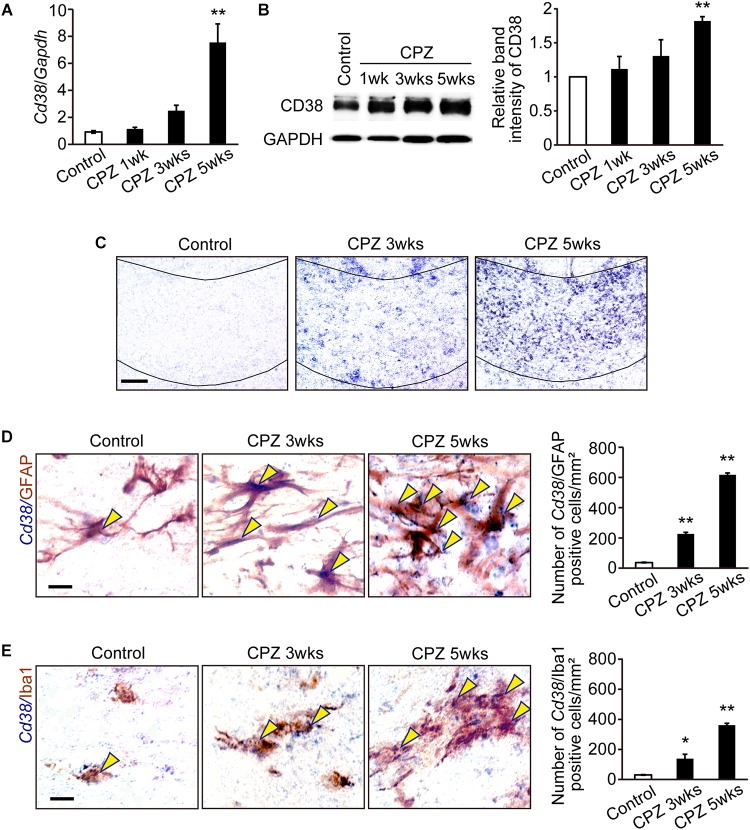
CD38 expression was increased in demyelinating lesions. **(A)** RT-qPCR analysis for *Cd38* mRNA expression in the CC after administration of CPZ for 1, 3, and 5 weeks *n* = 5. **(B)** Western blotting analyses of CD38 in the CC after different periods of CPZ administration. The graph depicts the relative optical density of CD38 normalized to the loading control glyceraldehyde 3-phosphate dehydrogenase (GAPDH) *n* = 5. **(C)** *In situ* hybridization analyses in the CC after administration of CPZ for different period using an antisense RNA probe to *Cd38*. Scale bar: 100 μm. **(D**,**E)** Double *in situ* hybridization-immunohistochemistry analyses in the CC after CPZ administration for 3 and 5 weeks with an antisense RNA probe to *Cd38* and antibodies against GFAP (upper) and Iba1 (lower). Yellow arrowheads indicate *Cd38*-expressing astrocytes or microglia. The graph depict the numbers of *Cd38*/GFAP- or *Cd38*/Iba1-positive cells in the CC after CPZ administration for 3 and 5 weeks, *n* = 4. Data are expressed as mean ± SEM. Scale bars: 20 μm. *P* values are determined by one-way ANOVA followed by Tukey-kramer test. ^∗^*p* < 0.05 and ^∗∗^*p* < 0.01 vs. control.

We first investigated the expression of CD38 in the CC in WT mice at different time points after CPZ administration. RT-qPCR revealed that the expression of CD38 mRNA was significantly increased 5 weeks after CPZ administration (Figure 1A). Western blotting also revealed that the expression of CD38 protein was gradually increased and reached a significantly higher level 5 weeks after CPZ administration when compared to that in the normal condition (Figure 1B). *In situ* hybridization confirmed the induction of CD38 mRNA in the CC 3–5 weeks after CPZ administration (Figure 1C). Furthermore, *in situ* hybridization-immunohistochemistry using cell type-specific antibodies revealed that the levels of CD38 transcripts were significantly increased both in GFAP-positive astrocytes and in Iba1-positive microglia (Figure 1D,E) 3–5 weeks after CPZ administration. Consistent with our recent report (Hattori et al., 2017), the number of CD38-positive cells among APC-positive OLs was much lower than that of GFAP-positive astrocytes (Supplementary Figure 1).

### Demyelination Was Attenuated, but Myelin Clearance Was Reduced in CD38 KO Mice

**FIGURE 2:**
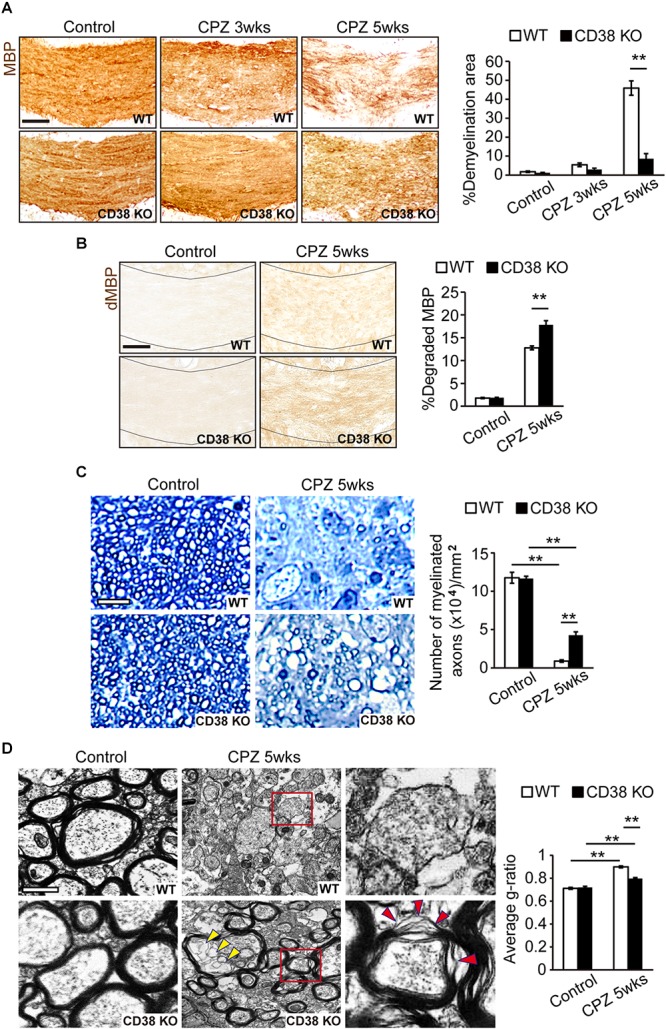
Demyelination was attenuated in CD38 KO mice. **(A)** Representative images of MBP immunohistochemistry in the CC of control and CPZ-administered (3 and 5 weeks) WT and CD38 KO mice. Scale bar: 200 μm. The graph shows densitometric analysis of the demyelinating areas in the CC of control and CPZ-administered (3 and 5 weeks) WT and CD38 KO mice, *n* = 4. **(B)** Representative images of dMBP immunohistochemistry in the CC of control and CPZ-administered (5 weeks) WT and CD38 KO mice. Scale bar: 200 μm. The graph shows densitometric analysis of the demyelinating areas in the CC of control and CPZ-administered (5 weeks) WT and CD38 KO mice, *n* = 4. **(C)** Toluidine blue staining of semi-thin resin sections of the CC of control and CPZ-administered (5 weeks) WT and CD38 KO mice. The graph shows the number of myelinated axons in the CC (*n* = 4 in each group). Scale bar: 5 μm. **(D)** Representative electron microscopic images of sagittal-sections of the CC of control and CPZ-administered (5 weeks) WT and CD38 KO mice. Right panels show higher magnification of the boxed areas in the middle panels. Yellow and red arrowheads in the lower panels indicate vacuoles and sheath breakdown in the myelinated axons, respectively. The graph shows g-ratio (axon diameter/fiber diameter) of the myelin sheath (*n* = 100 axons in each group). Scale bar: 1 μm. Data are expressed as mean ± SEM. *P* values are determined by two-way ANOVA followed by Scheffe’s *F* test. ^∗∗^*p* < 0.01 between two conditions.

To investigate the role of CD38 in the process of demyelination, CPZ was administered to both WT, and CD38 KO mice for 5 weeks. Demyelination was analyzed by immunohistochemistry for MBP. The normal myelin pattern was observed in the CC of mice from both genotypes under physiological conditions (Figure 2A). In contrast, demyelination was observed 3 weeks and then strongly increased 5 weeks after CPZ administration in WT mice (upper panels in Figure 2A). Interestingly, demyelination was significantly decreased in CD38 KO mice 5 weeks after CPZ administration (lower panels in Figure 2A). To evaluate whether preserved myelin in CD38 KO mice was functionally intact or degraded, we performed immunohistochemistry for dMBP (Figure 2B). The degraded myelin was increased in both genotypes, but the level was higher in CD38 KO mice than in WT mice 5 weeks after CPZ administration, suggesting that the preserved myelin in CD38 KO mice includes the degraded one. Toluidine Blue staining of semi-thin sections of the CC confirmed a strong reduction in the number of myelinated axons in both genotypes 5 weeks after CPZ administration, but the level was milder in CD38 KO mice than in WT mice (Figure 2C). Furthermore, ultrastructure analyses using electron microscopy revealed nearly complete demyelination in WT mice (Upper panels in Figure 2D), while relatively preserved myelin, and improved g-ratio in CD38 KO mice (lower panels in Figure 2D and the graph) 5 weeks after CPZ administration. However, the majority of the remaining myelin in CD38 KO mice showed an altered myelin structure. The myelin sheaths in CD38 KO mice displayed splits/breakdown and vacuoles (red and yellow arrowheads, respectively, in Figure 2D). Additionally, activated microglia with many phagocytozed materials, probably degraded myelin, were often observed in WT mice, but not in CD38 KO mice, after CPZ administration (Supplementary Figure 2).

### Axonal Damage Was Attenuated in CD38 KO Mice

**FIGURE 3:**
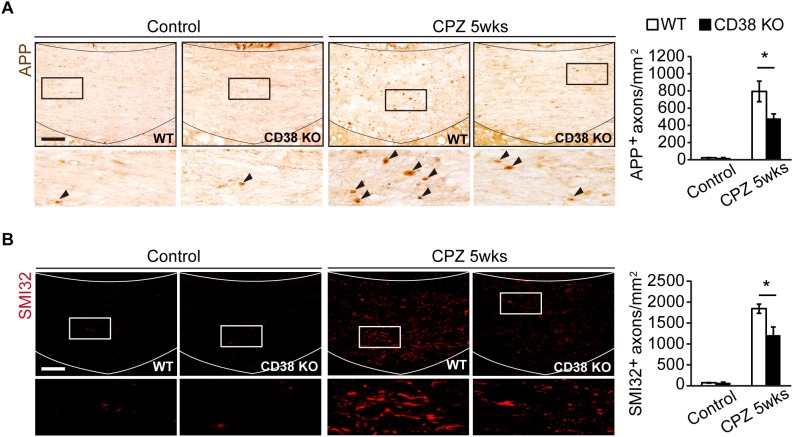
Axonal damage was attenuated in CD38 KO mice. **(A)** Representative images of APP immunohistochemistry in the CC of control and CPZ-administered (5 weeks) WT and CD38 KO mice. Lower panels show higher magnification of the boxed areas in the upper panels. Arrowheads indicate APP-positive cells. The graph represents the number of APP-positive axons in the CC of control and CPZ-administered (5 weeks) WT and CD38 KO mice, *n* = 4. **(B)** Representative images of SMI-32 immunohistochemistry in the CC of control and CPZ-administered (5 weeks) WT and CD38 KO mice. The graph represents the number of SMI-32-positive axons in the CC of control and CPZ-administered (5 weeks) WT and CD38 KO mice, *n* = 4. Data are expressed as mean ± SEM. *P* values are determined by two-way ANOVA followed by Scheffe’s *F* test. ^∗^*p* < 0.05 between two conditions. Scale bars: 100 μm.

It has been reported that, together with extensive demyelination, CPZ administration causes axonal damage which is characterized by axonal swelling and bulb-like formation (Piaton et al., 2010). These structures include deposits of APP (Petkovic et al., 2016) and dephosphorylated neurofilament (Herrero-Herranz et al., 2008; Havranek et al., 2017). To analyze the extent of axonal damage after CPZ administration, we performed immunohistochemistry using anti-APP antibody (Figure 3A) and anti-SMI-32 antibody (Figure 3B), the latter recognizes non-phosphorylated forms of neurofilament H. More APP- and SMI-32-positive deposits were observed in WT mice than in CD38 KO mice after CPZ administration. These results suggest that CPZ-induced axonal damage was attenuated in CD38 KO mice.

### OL Repopulation Was Impaired in CD38 KO Mice

**FIGURE 4:**
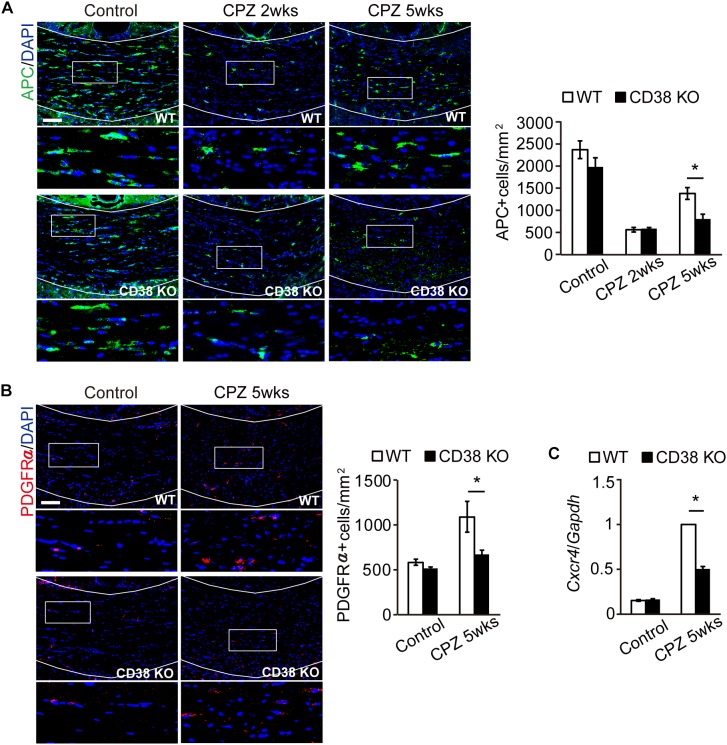
OL repopulation was impaired in CD38 KO mice. **(A**,**B)** Representative images of APC **(A)** and PDGFRα **(B)** immunohistochemistry in the CC of control and CPZ-administered WT and CD38 KO mice. Nuclei were counterstained with DAPI. Lower panels show higher magnification of the boxed areas in the upper panels. The graph represents the number of APC- or PDGFRα-positive cells in the CC of control and CPZ-administered (2 and 5 weeks) WT and CD38 KO mice, *n* = 4. **(C)** RT-qPCR analysis for *Cxcr4* expression in the CC of control and CPZ-administered (5 weeks) WT and CD38 KO mice, *n* = 4. Data are expressed as mean ± SEM. *P* values are determined by two-way ANOVA followed by Scheffe’s *F* test. ^∗^*p* < 0.05 between two conditions. Scale bars: 100 μm.

CPZ administration induces selective apoptosis in the mature OLs early on (first 2 weeks), followed by the accumulation of OPCs to the demyelinating lesion, and differentiation into the new mature OLs, which is a critical step for remyelination (Praet et al., 2014). We thus analyzed the status of APC-expressing mature OLs and PDGFR-expressing OPCs during CPZ administration. Immunohistochemistry revealed that, although the initial loss of mature OLs 2 weeks after CPZ administration was observed to a similar level in both genotypes (Figure 4A,B), the number of OPCs (Figure 4B), and mature OLs (Figure 4A) were significantly lower in CD38 KO mice than in WT mice 5 weeks after CPZ administration. Consistent with these results, RT-qPCR analysis revealed that the expression of CXCR4, which is induced in the OPCs and promote their maturation and remyelination after CPZ administration (Patel et al., 2010), was significantly lower in CD38 KO mice 5 weeks after CPZ administration (Figure 4C). These results suggest suppression of the OL repopulation in CD38 KO mice after CPZ administration.

### Glial Activation Was Attenuated in CD38 KO Mice

**FIGURE 5:**
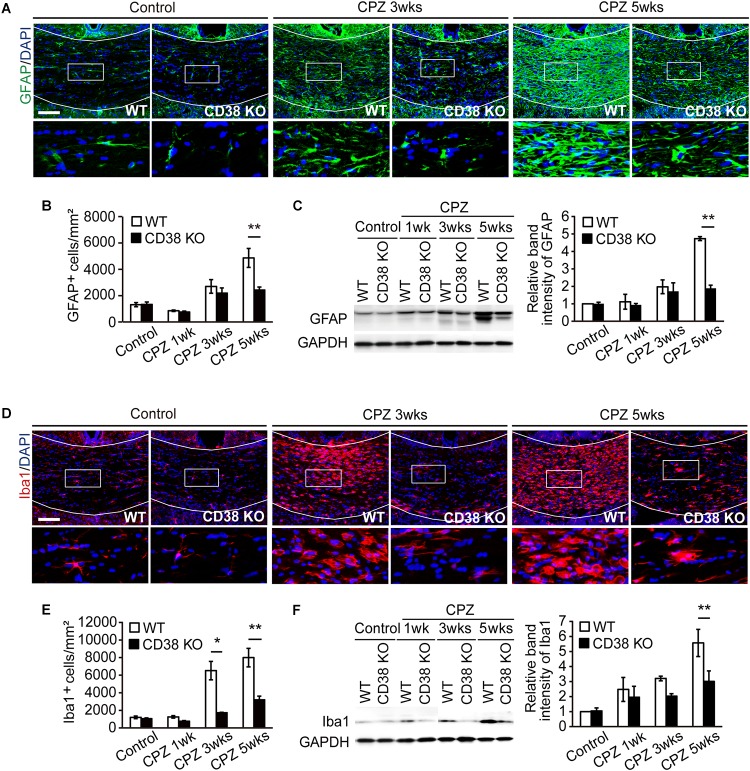
Glial activation was attenuated in CD38 KO mice. **(A)** Representative images of GFAP immunohistochemistry in the CC of control and CPZ-administered (3 and 5 weeks) WT and CD38 KO mice. Nuclei were counterstained with DAPI. Lower panels show higher magnification of the boxed areas in the upper panels. **(B)** The Graph represents the number of GFAP-positive astrocytes in the CC of control and CPZ-administered (1, 3, and 5 weeks) WT and CD38 KO mice, *n* = 4. **(C)** Western blotting analyses of GFAP in the CC of WT and CD38 KO mice after different periods of CPZ administration. The graph depicts the relative optical density of GFAP normalized to the loading control GAPDH *n* = 5. **(D)** Representative images of Iba1 immunohistochemistry in the CC of control and CPZ-administered (3 and 5 weeks) WT and CD38 KO mice. Nuclei were counterstained with DAPI. Lower panels show higher magnification of the boxed areas in the upper panels. **(E)** The graph represents the number of Iba1-positive microglia in the CC of control and CPZ-administered (1, 3, and 5 weeks) WT and CD38 KO mice *n* = 5. **(F)** Western blotting analyses of Iba1 in the CC of WT and CD38 KO mice after different periods of CPZ administration. The graph depicts the relative optical density of Iba1 normalized to the loading control GAPDH *n* = 5. Data are expressed as mean ± SEM. *P* values are determined by two-way ANOVA followed by Scheffe’s *F* test. ^∗^*p* < 0.05 and ^∗∗^*p* < 0.01 between two conditions. Scale bars: 100 μm.

As demyelination is accompanied by extensive astroglial and microglial responses that greatly influence the extent of further demyelination and remyelination (Gudi et al., 2014), the status of glial cells was analyzed by immunohistochemistry, and western blot for GFAP and Iba1. The numbers of GFAP-positive cells and the expression levels of GFAP protein were increased after CPZ administration in WT mice, but were significantly lower in CD38 KO mice (Figure 5A–C). Similarly, the numbers of Iba1-positive cells and the expression levels of Iba1 protein were increased after CPZ administration, but were significantly lower in CD38 KO mice (Figure 5D–F). These data indicate that deletion of CD38 suppresses CPZ-induced glial activation.

### Inflammatory Responses Were Reduced in CD38 KO Mice

**FIGURE 6:**
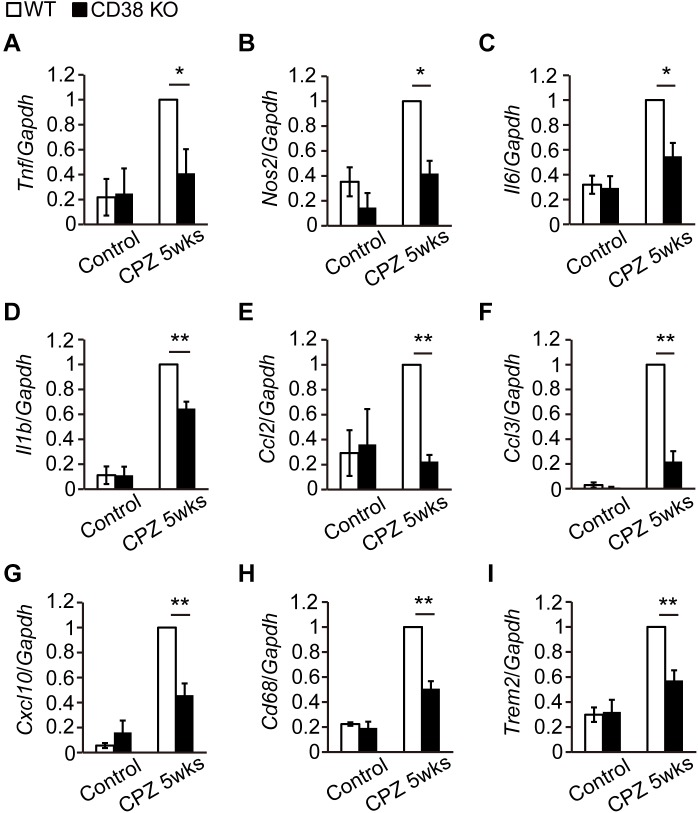
Expression levels of inflammatory genes were decreased in CD38 KO mice. **(A–I)** RT-qPCR analysis for the expression of inflammatory genes in the CC of control and CPZ-administered (5 weeks) WT and CD38 KO mice *n* = 5. Data are expressed as mean ± SEM. *P* values are determined by two-way ANOVA followed by Scheffe’s *F* test. ^∗^*p* < 0.05 and ^∗∗^*p* < 0.01 between two conditions.

Activated astrocytes and microglia produce a battery of inflammatory cytokines/chemokines (Gudi et al., 2014) and subsequently harm OLs and axons. RT-qPCR analysis revealed that the expressions of genes such as *Tnf, Il1b, Nos2, Ccl2, Ccl3*, and *Cxcl10* were increased after CPZ administration (Figure 6A–G), but their levels were lower in CD38 KO mice, suggesting that deletion of CD38 suppresses the induction of inflammatory genes. Similarly, the expression levels of phagocytosis-associated genes such as *Trem2* (Triggering Receptor Expressed on Myeloid cells) and *Cd68* genes were significantly lower in CD38 KO mice after CPZ administration (Figure 6H,I).

### Silencing/Deletion of *Cd38* Gene Suppressed the Activation of Astrocytes and Microglia *in vitro*

**FIGURE 7:**
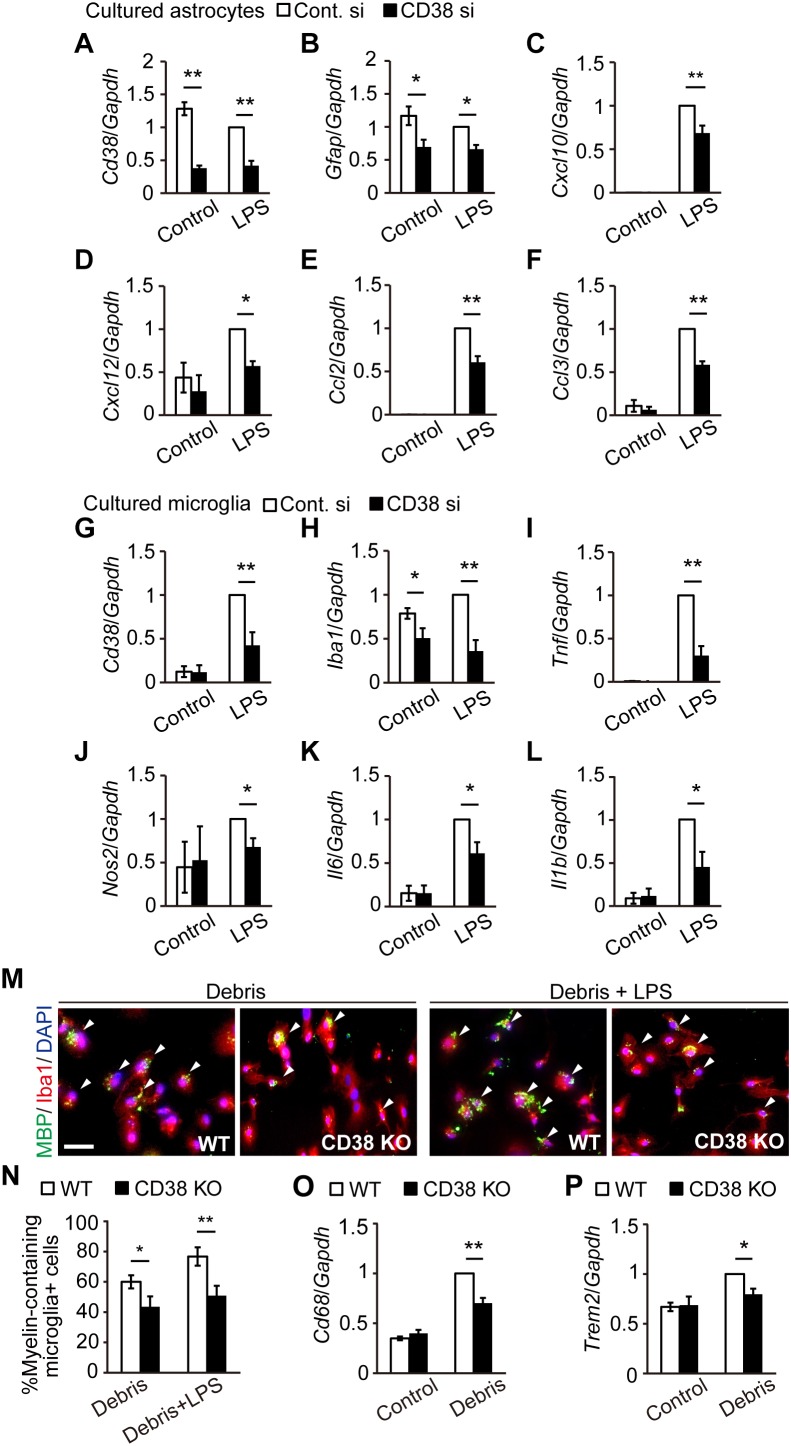
Silencing/deletion of *Cd38* gene reduced the activation of astrocytes and microglia *in vitro.* **(A–F)** Primary astrocyte cultures were transfected with CD38 siRNA or control siRNA at 4 and 6 days *in vitro* (DIV) and stimulated with LPS at 8 DIV. Total RNA was prepared from cells 6 h after treatment with LPS. The expression levels of *Cd38, Gfap, Cxcl10, Cxcl12*, *Ccl2*, and *Ccl3* transcripts were determined using RT-qPCR *n* = 7. **(G–L)** Primary microglia cultures were transfected with CD38 siRNA or control siRNA at 1 DIV and treated with LPS at 2 DIV. Total RNA was prepared from cells 6 h after treatment with LPS. The expression levels of *Cd38, Iba1, Tnf, Nos2, Il6*, and *Il1β* transcripts were determined using RT-qPCR *n* = 5. **(M–P)** Evaluation of phagocytois. Primary microglia cultures of WT and CD38 KO mice were treated with LPS and myelin debris for 24 h. **(M)** Cells were stained with anti-Iba1 and -MBP antibodies. Arrowheads indicate phagocytic microglial containing MBP inside the cell body. Scale bar: 20 μm. **(N)** The graph depicts percentage of myelin-containing microglia *n* = 5. **(O**,**P)** The expression levels of *Cd68* and *Trem2* transcripts were determined by RT-qPCR *n* = 5. All data are expressed as mean ± SEM. All *P* values are determined by two-way ANOVA followed by Scheffe’s *F* test. ^∗^*p* < 0.05 and ^∗∗^*p* < 0.01 between two conditions.

To determine whether CD38 regulates glial activation cell-autonomously, we analyzed the expression of glial markers and cytokines/chemokines in both cultured astrocytes (Figure 7A–F) and microglia (Figure 7G–L) after transfecting them with CD38 siRNA or control RNA. In astrocytes, the expression levels of *Cd38* and *Gfap* were significantly reduced following the silencing of *Cd38* gene, both in the absence and presence of lipopolysaccharide (LPS), as we recently described (Hattori et al., 2017). Although LPS strongly induced astrocyte-related chemokines such as *Cxcl10, Cxcl12, Ccl2*, and *Ccl3* in both genotypes, the levels were significantly lower in CD38 siRNA-transfected cells (Figure 7B–F). These results indicate that CD38 regulates the activation of astrocytes cell-autonomously.

In microglia, LPS also induced *Cd38* and glia-related inflammatory genes, such as *Nos2* and those coding for cytokines in both genotypes, while the levels were significantly lower in CD38 siRNA-transfected microglia (Figure 7G,I–L). The expression of *Iba1* was also reduced by silencing CD38 both in the absence and presence of LPS (Figure 7H). CD38 KO microglia had a similar phenotype to microglia subjected to CD38 silencing (Supplementary Figures 3A–F). Furthermore, CD38 KO microglia exhibited lower phagocytic activity (Figure 7M,N) and lower levels of expressions of phagocytosis-associated molecules (Figure 7O,P). These results indicate that CD38 also regulates the activation of microglia cell-autonomously.

### NAD^+^ Suppressed the Activation of Astrocytes and Microglia

**FIGURE 8:**
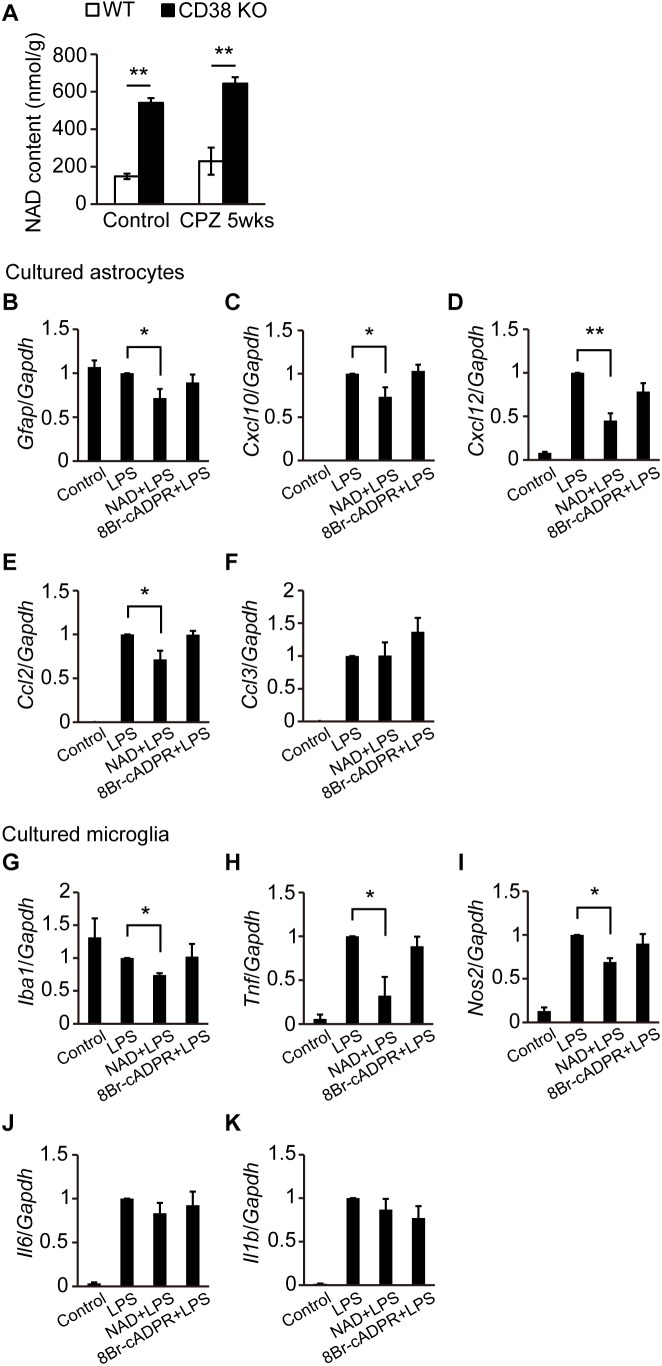
NAD^+^ suppressed the activation of astrocytes and microglia. **(A)** Determination of NAD levels in the CC of control and CPZ-administered (5 weeks) WT and CD38 KO mice, *n* = 4. *P* values are determined by two-way ANOVA followed by Scheffe’s *F* test. ^∗∗^*p* < 0.01 between two conditions. **(B–F)** Primary astrocyte cultures were treated with NAD^+^ (200 μM) or 8Br-cADPR (20 μM) for 5 days (4–9 DIV) and then stimulated with LPS at 9 DIV *n* = 6. *P* values are determined by one-way ANOVA followed by Tukey-kramer test. ^∗^*p* < 0.05 and ^∗∗^*p* < 0.01 between two conditions. **(G–K)** Microglia cultures were treated with NAD^+^ (200 μM) or 8Br-cADPR (20 μM) for 1 day (1–2 DIV), and then stimulated with LPS at 2 DIV *n* = 6. *P* values are determined by one-way ANOVA followed by Tukey-kramer test. ^∗^*p* < 0.05 between two conditions. In **(B–K)**, total RNA was prepared from cells 6 h after treatment with LPS. The expression levels of *Gfap, Iba1*, and proinflammatory molecule transcripts were determined by RT-qPCR. All data are expressed as mean ± SEM.

Since we and other groups have reported that CD38 KO mice exhibit increased NAD^+^ levels in the brain (Jin et al., 2007; Hattori et al., 2017), we measured NAD (NAD^+^ + NADH) concentrations in the CC of WT and CD38 KO mice administered CPZ as well as those not administered CPZ. Consistent with the results of our recent study, NAD levels were significantly higher in CD38 KO mice than in WT mice in the normal condition (control). Although CPZ administration for 5 weeks slightly increased the NAD level in mice of both genotypes, CD38 KO mice still exhibited significantly higher levels of NAD than WT mice (Figure 8A). We also investigated the effects of NAD^+^ and cADPR, which are the substrate and product of CD38, respectively, on the activation of astrocytes and microglia. Pretreatment of cultured astrocytes or microglia with NAD^+^, but not with 8Br-cADPR (a cADPR antagonist), reduced the expression levels of *Gfap, Cxcl10, Cxcl12*, and *Ccl2* in astrocytes (Figure 8B–F), and those of *Iba1* and some inflammatory genes such as *Tnf* and *Nos2* in microglia (Figure 8G–K) after LPS stimulation. These results suggest that deletion of the *Cd38 gene* suppresses the activation of astrocytes and microglia by increasing NAD^+^ levels.

## Discussion

In the current study, we examined the role of CD38 in CPZ-induced demyelination model in mice. CD38 expression was enhanced in both astrocytes and microglia after CPZ administration. The deletion of CD38 ameliorated demyelination and neurodegeneration, while it suppressed myelin clearance and subsequent OL repopulation. CD38 deficiency, or addition of NAD^+^ suppressed activation of both astrocytes and microglia, and inflammatory responses in those cells. These results suggest that CD38 in the glial cells regulates neuroinflammation by controlling NAD^+^ level in the brain, and affects both of CPZ-induced demyelination and subsequent OL remodeling.

In a recent report, Herrmann et al. (2016) demonstrated that CD38 was crucially involved in the pathology of EAE. CD38 expression was strongly elevated both in the encephalitogenic lymph node (LN) cells and in the CNS-infiltrating cells after induction of EAE. Furthermore, deletion of CD38 ameliorated clinical symptoms, and its effect was associated with reduced levels of both antibody production and T-cell response in CD38 KO cells after administration of myelin oligodendrocyte glycoprotein (MOG) 1-125. In contrast to their report, our study demonstrated both beneficial and detrimental effects of CD38 deletion in a different MS model, CPZ-induced demyelination model in mice, which lacks an autoimmune lymphocytic response in its pathology. The expression of CD38 was also increased in the CPZ-induced demyelinating region (Figure 1A–C), and consistent with the observations in EAE, deletion of CD38 ameliorated both demyelination (Figure 2A,C) and neurodegeneration (Figure 3A,B), while underlying mechanisms may be different. In our model, CD38 played critical roles in the glial activation and subsequent neuroinflammation both in mice (Figure 5, 6A–G) and in cultured astroglial and microglial cells (Figure 7A–L and Supplementary Figure 3), which caused secondary damages to OLs and neurons through the production of toxic cytokines and chemokines such as TNF-α, IL-1β, iNOS, CCL2, CCL3, and CXCL10 (Selmaj and Raine, 1988; Merrill et al., 1993; McMahon et al., 2001; Skripuletz et al., 2013). We speculate that it will be quite interesting and important to study more detail of the role of glial CD38 in the settings of EAE and MS.

In the present study, we also observed the reduced levels of the clearance of degraded myelin (Figure 2B,D) and oligodendrocyte repopulation (Figure 4A–C) in CD38 KO mice 5 weeks after CPZ administration. Consistent with these results, the phagocytic activity (Figure 7M,N and Supplementary Figures 2A,B) and the expression of phagocytosis-associated genes such as *Cd68* and *Trem2* (Figure 6H,I, 7O,P) were reduced in CD38 KO mice and in CD38 KO mice-derived microglia. As microglial phagocytosis of the degraded myelin is essential for the proper OPC maturation into OLs after demyelination (Petkovic et al., 2016), our findings suggest that CD38-mediated glial activation, and phagocytosis may also play a critical role in the process of remyelination. However, it is not clear yet how the numbers of both OPCs and OLs were reduced in CD38 KO mice 5 weeks after CPZ administration (Figure 4A–C). Our previous report demonstrated that astrocytic CD38 facilitated the maturation of OPCs, but not their own proliferation in the postnatal developing periods (Hattori et al., 2017). Further studies are required to clarify this point.

Herrmann et al. (2016) also demonstrated in their report that Sphingosine 1-phosphate (S1P)-receptor modulator FTY720 (fingolimod), which is the first oral therapeutic agent for MS and exerts its function, at least in part, by inhibiting lymphocyte trafficking from secondary lymphoid organs, effectively suppressed EAE severity and reduced the level of CD38 expression in the lymph nodes. Interestingly, S1P receptors are also highly expressed in the brain cells including astrocytes (Van Doorn et al., 2010; Choi et al., 2011). It was previously reported that FTY720 ameliorated demyelination and neurodegeneration in CPZ-induced demyelination model, while it failed to enhance remyelination *in vivo* (Kim et al., 2011; Slowik et al., 2015). Although our preliminary results have revealed no significant differences between WT and CD38 KO mice in the expression levels of S1P and AKT2, a downstream molecule in SIP signaling, 5 weeks after CPZ administration, further analysis may clarify the possible link between S1P and CD38 pathways in the glia-mediated regulation of demyelination and neurodegeneration.

It was also reported that nicotinamide, which is a precursor of CD38 substrate NAD^+^, reduced demyelination, axonal degeneration, and infiltration of CD4^+^ T-cells in EAE (Kaneko et al., 2006). Furthermore, NAD^+^ has been shown to reverse the progression of EAE by regulating CD4^+^ T-cell differentiation and apoptosis (Tullius et al., 2014). These studies indicate that NAD^+^ has beneficial effects on MS pathology. In the current study, we demonstrated the suppressing effect of NAD^+^ on the glial activation, as well as inflammatory responses *in vitro*, and the higher level of NAD in the CD38 KO brains in both of the control and CPZ-administrated conditions. We speculate that NAD^+^ also has both beneficial and detrimental effects in the process of demyelination, depending on the situations such as acute inflammatory phase and chronic neurodegenerating phase. Although the molecular mechanism of NAD^+^-mediated regulation of glial activity is still unclear, a NAD^+^-dependent deacetylase sirtuin2 (SIRT2) in microglia may be involved in this process, as previously described (Pais et al., 2013). Furthermore, since brain NAD^+^ level can be increased by administration of NAD^+^ precursors such as nicotinamide riboside and nicotinamide mononucleotide (Gong et al., 2013), it will be intriguing to test these molecules in CPZ-induced demyelination model.

In conclusion, we identified novel effects of CD38 and NAD^+^ on demyelination and neuroinflammation using CPZ-induced demyelination model. CD38 and NAD^+^ in glial cells may have both beneficial and detrimental effects, and further studies to regulate the balance of these effects will make them potential targets for therapeutic intervention of MS and other demyelinating diseases.

## Data Availability

All datasets generated for this study are included in the manuscript and/or the Supplementary Files.

## Ethics Statement

All animal experiments were performed in accordance with the guideline and approved by the Animal Care and Use Committee of Kanazawa University (AP-143305).

## Author Contributions

TH and OH designed the experiments. JR, TH, and YS conducted the studies. HI, MT-I, NO, and TL assisted with the experiments and provided the intellectual input. YY, AS, HO, and HH supervised the study. JR, TH, HO, HH, YK, and OH interpreted the data and wrote the manuscript.

## Conflict of Interest Statement

The authors declare that the research was conducted in the absence of any commercial or financial relationships that could be construed as a potential conflict of interest.

## References

[B1] Akimoto N. Kamiyama Y. Yamafuji M. Fujita K. Seike T. Kido M. (2013). Immunohistochemistry of CD38 in different cell types in the hypothalamus and pituitary of male mice. *Am. Sci. Pub.* 2 54–61. 10.1166/msr.2013.1021

[B2] Blakemore W. F. (1972). Observations on oligodendrocyte degeneration, the resolution of status spongiosus and remyelination in cuprizone intoxication in mice. *J. Neurocytol.* 1 413–426. 10.1007/bf01102943 8530973

[B3] Bond L. Lusher D. Williams I. Butler H. (2014). Friends or foes? Relational dissonance and adolescent psychological wellbeing. *PLoS One* 9:e83388. 10.1371/journal.pone.0083388 24498257 PMC3911895

[B4] Cao L. He C. (2013). Polarization of macrophages and microglia in inflammatory demyelination. *Neurosci. Bull.* 29 189–198. 10.1007/s12264-013-1324-1320 23558588 PMC5561884

[B5] Choi J. W. Gardell S. E. Herr D. R. Rivera R. Lee C. W. Noguchi K. (2011). FTY720 (fingolimod) efficacy in an animal model of multiple sclerosis requires astrocyte sphingosine 1-phosphate receptor 1 (S1P1) modulation. *Proc. Natl. Acad. Sci. U.S.A.* 108 751–756. 10.1073/pnas.1014154108 21177428 PMC3021041

[B6] Clarner T. Janssen K. Nellessen L. Stangel M. Skripuletz T. Krauspe B. (2015). CXCL10 triggers early microglial activation in the cuprizone model. *J. Immunol.* 194 3400–3413. 10.4049/jimmunol.1401459 25725102

[B7] Compston A. Coles A. (2008). Multiple sclerosis. *Lancet* 372 1502–1517. 10.1016/S0140-6736(08)61620-61627 18970977

[B8] Fan H. B. Chen L. X. Qu X. B. Ren C. L. Wu X. X. Dong F. X. (2017). Transplanted miR-219-overexpressing oligodendrocyte precursor cells promoted remyelination and improved functional recovery in a chronic demyelinated model. *Sci. Rep.* 7:41407. 10.1038/srep41407 28145507 PMC5286453

[B9] Goebbels S. Oltrogge J. H. Kemper R. Heilmann I. Bormuth I. Wolfer S. (2010). Elevated phosphatidylinositol 3,4,5-trisphosphate in glia triggers cell-autonomous membrane wrapping and myelination. *J. Neurosci.* 30 8953–8964. 10.1523/JNEUROSCI.0219-10.2010 20592216 PMC6632897

[B10] Gong B. Pan Y. Vempati P. Zhao W. Knable L. Ho L. (2013). Nicotinamide riboside restores cognition through an upregulation of proliferator-activated receptor-gamma coactivator 1alpha regulated beta-secretase 1 degradation and mitochondrial gene expression in Alzheimer’s mouse models. *Neurobiol. Aging* 34 1581–1588. 10.1016/j.neurobiolaging.2012.12.005 23312803 PMC3632303

[B11] Gudi V. Gingele S. Skripuletz T. Stangel M. (2014). Glial response during cuprizone-induced de- and remyelination in the CNS: lessons learned. *Front. Cell Neurosci.* 8:73. 10.3389/fncel.2014.00073 24659953 PMC3952085

[B12] Hattori T. Baba K. Matsuzaki S. Honda A. Miyoshi K. Inoue K. (2007). A novel DISC1-interacting partner DISC1-binding zinc-finger protein: implication in the modulation of DISC1-dependent neurite outgrowth. *Mol. Psychiatry* 12 398–407. 10.1038/sj.mp.4001945 17389905

[B13] Hattori T. Kaji M. Ishii H. Jureepon R. Takarada-Iemata M. Minh Ta H. (2017). CD38 positively regulates postnatal development of astrocytes cell-autonomously and oligodendrocytes non-cell-autonomously. *Glia* 65 974–989. 10.1002/glia.23139 28295574

[B14] Hattori T. Shimizu S. Koyama Y. Emoto H. Matsumoto Y. Kumamoto N. (2014). DISC1 (disrupted-in-schizophrenia-1) regulates differentiation of oligodendrocytes. *PLoS One* 9:e88506. 10.1371/journal.pone.0088506 24516667 PMC3917910

[B15] Hattori T. Shimizu S. Koyama Y. Yamada K. Kuwahara R. Kumamoto N. (2010). DISC1 regulates cell-cell adhesion, cell-matrix adhesion and neurite outgrowth. *Mol. Psychiatry* 15 798–809. 10.1038/mp.2010.60 20479754

[B16] Havranek T. Lestanova Z. Mravec B. Strbak V. Bakos J. Bacova Z. (2017). Oxytocin modulates expression of neuron and glial markers in the Rat hippocampus. *Folia Biol.* 63 91–97. 28805558 10.14712/fb2017063030091

[B17] Herrero-Herranz E. Pardo L. A. Gold R. Linker R. A. (2008). Pattern of axonal injury in murine myelin oligodendrocyte glycoprotein induced experimental autoimmune encephalomyelitis: implications for multiple sclerosis. *Neurobiol. Dis.* 30 162–173. 10.1016/j.nbd.2008.01.001 18342527

[B18] Herrmann M. M. Barth S. Greve B. Schumann K. M. Bartels A. Weissert R. (2016). Identification of gene expression patterns crucially involved in experimental autoimmune encephalomyelitis and multiple sclerosis. *Dis. Model. Mech.* 9 1211–1220. 10.1242/dmm.025536 27519689 PMC5087830

[B19] Jin D. Liu H. X. Hirai H. Torashima T. Nagai T. Lopatina O. (2007). CD38 is critical for social behaviour by regulating oxytocin secretion. *Nature* 446 41–45. 10.1038/nature05526 17287729

[B20] Kaneko S. Wang J. Kaneko M. Yiu G. Hurrell J. M. Chitnis T. (2006). Protecting axonal degeneration by increasing nicotinamide adenine dinucleotide levels in experimental autoimmune encephalomyelitis models. *J. Neurosci.* 26 9794–9804. 10.1523/JNEUROSCI.2116-06.2006 16988050 PMC6674451

[B21] Kato I. Yamamoto Y. Fujimura M. Noguchi N. Takasawa S. Okamoto H. (1999). CD38 disruption impairs glucose-induced increases in cyclic ADP-ribose, [Ca2+]i, and insulin secretion. *J. Biol. Chem.* 274 1869–1872. 10.1074/jbc.274.4.1869 9890936

[B22] Kim H. J. Miron V. E. Dukala D. Proia R. L. Ludwin S. K. Traka M. (2011). Neurobiological effects of sphingosine 1-phosphate receptor modulation in the cuprizone model. *FASEB J.* 25 1509–1518. 10.1096/fj.10-173203 21248243 PMC3079302

[B23] Kou W. Banerjee S. Eudy J. Smith L. M. Persidsky R. Borgmann K. (2009). CD38 regulation in activated astrocytes: implications for neuroinflammation and HIV-1 brain infection. *J. Neurosci. Res.* 87 2326–2339. 10.1002/jnr.22060 19365854

[B24] Lassmann H. Van Horssen J. Mahad D. (2012). Progressive multiple sclerosis: pathology and pathogenesis. *Nat. Rev. Neurol.* 8 647–656. 10.1038/nrneurol.2012.168 23007702

[B25] Lee H. C. (2001). Physiological functions of cyclic ADP-ribose and NAADP as calcium messengers. *Annu. Rev. Pharmacol. Toxicol.* 41 317–345. 10.1146/annurev.pharmtox.41.1.317 11264460

[B26] Levy A. Bercovich-Kinori A. Alexandrovich A. G. Tsenter J. Trembovler V. Lund F. E. (2009). CD38 facilitates recovery from traumatic brain injury. *J. Neurotrauma* 26 1521–1533. 10.1089/neu.2008.0746 19257806 PMC2864472

[B27] Levy A. Blacher E. Vaknine H. Lund F. E. Stein R. Mayo L. (2012). CD38 deficiency in the tumor microenvironment attenuates glioma progression and modulates features of tumor-associated microglia/macrophages. *Neuro. Oncol.* 14 1037–1049. 10.1093/neuonc/nos121 22700727 PMC3408254

[B28] Malavasi F. Deaglio S. Funaro A. Ferrero E. Horenstein A. L. Ortolan E. (2008). Evolution and function of the ADP ribosyl cyclase/CD38 gene family in physiology and pathology. *Physiol. Rev.* 88 841–886. 10.1152/physrev.00035.2007 18626062

[B29] McMahon E. J. Cook D. N. Suzuki K. Matsushima G. K. (2001). Absence of macrophage-inflammatory protein-1alpha delays central nervous system demyelination in the presence of an intact blood-brain barrier. *J. Immunol.* 167 2964–2971. 10.4049/jimmunol.167.5.2964 11509646

[B30] Merrill J. E. Ignarro L. J. Sherman M. P. Melinek J. Lane T. E. (1993). Microglial cell cytotoxicity of oligodendrocytes is mediated through nitric oxide. *J. Immunol.* 151 2132–2141. 8102159

[B31] Pais T. F. Szego E. M. Marques O. Miller-Fleming L. Antas P. Guerreiro P. (2013). The NAD-dependent deacetylase sirtuin 2 is a suppressor of microglial activation and brain inflammation. *EMBO J.* 32 2603–2616. 10.1038/emboj.2013.200 24013120 PMC3791374

[B32] Pasquini L. A. Calatayud C. A. Bertone Una A. L. Millet V. Pasquini J. M. Soto E. F. (2007). The neurotoxic effect of cuprizone on oligodendrocytes depends on the presence of pro-inflammatory cytokines secreted by microglia. *Neurochem. Res.* 32 279–292. 10.1007/s11064-006-9165-9160 17063394

[B33] Patel J. R. Mccandless E. E. Dorsey D. Klein R. S. (2010). CXCR4 promotes differentiation of oligodendrocyte progenitors and remyelination. *Proc. Natl. Acad. Sci. U.S.A.* 107 11062–11067. 10.1073/pnas.1006301107 20534485 PMC2890706

[B34] Petkovic F. Campbell I. L. Gonzalez B. Castellano B. (2016). Astrocyte-targeted production of interleukin-6 reduces astroglial and microglial activation in the cuprizone demyelination model: implications for myelin clearance and oligodendrocyte maturation. *Glia* 64 2104–2119. 10.1002/glia.23043 27535761

[B35] Piaton G. Gould R. M. Lubetzki C. (2010). Axon-oligodendrocyte interactions during developmental myelination, demyelination and repair. *J. Neurochem.* 114 1243–1260. 10.1111/j.1471-4159.2010.06831.x 20524961

[B36] Praet J. Guglielmetti C. Berneman Z. Van Der Linden A. Ponsaerts P. (2014). Cellular and molecular neuropathology of the cuprizone mouse model: clinical relevance for multiple sclerosis. *Neurosci. Biobehav. Rev.* 47 485–505. 10.1016/j.neubiorev.2014.10.004 25445182

[B37] Raposo C. Nunes A. K. Luna R. L. Araujo S. M. Da Cruz-Hofling M. A. Peixoto C. A. (2013). Sildenafil (Viagra) protective effects on neuroinflammation: the role of iNOS/NO system in an inflammatory demyelination model. *Mediators Inflamm.* 2013:321460. 10.1155/2013/321460 23970812 PMC3736464

[B38] Remington L. T. Babcock A. A. Zehntner S. P. Owens T. (2007). Microglial recruitment, activation, and proliferation in response to primary demyelination. *Am. J. Pathol.* 170 1713–1724. 10.2353/ajpath.2007.060783 17456776 PMC1854965

[B39] Selmaj K. W. Raine C. S. (1988). Tumor necrosis factor mediates myelin and oligodendrocyte damage in vitro. *Ann. Neurol.* 23 339–346. 10.1002/ana.410230405 3132891

[B40] Shibata K. Murata K. (1986). Blood NAD as an index of niacin nutrition. *Nutr. Int.* 2 177–181. 2621487

[B41] Skripuletz T. Hackstette D. Bauer K. Gudi V. Pul R. Voss E. (2013). Astrocytes regulate myelin clearance through recruitment of microglia during cuprizone-induced demyelination. *Brain* 136 147–167. 10.1093/brain/aws262 23266461

[B42] Slowik A. Schmidt T. Beyer C. Amor S. Clarner T. Kipp M. (2015). The sphingosine 1-phosphate receptor agonist FTY720 is neuroprotective after cuprizone-induced CNS demyelination. *Br. J. Pharmacol.* 172 80–92. 10.1111/bph.12938 25220526 PMC4280969

[B43] Song S. Y. Kato C. Adachi E. Moriya-Sato A. Inagawa-Ogashiwa M. Umeda R. (2007). Expression of an acyl-CoA synthetase, lipidosin, in astrocytes of the murine brain and its up-regulation during remyelination following cuprizone-induced demyelination. *J. Neurosci. Res.* 85 3586–3597. 10.1002/jnr.21456 17722065

[B44] Takasawa S. Nata K. Yonekura H. Okamoto H. (1993). Cyclic ADP-ribose in insulin secretion from pancreatic beta cells. *Science* 259 370–373. 10.1126/science.8420005 8420005

[B45] Tullius S. G. Biefer H. R. Li S. Trachtenberg A. J. Edtinger K. Quante M. (2014). NAD^+^ protects against EAE by regulating CD4^+^ T-cell differentiation. *Nat. Commun.* 5:5101. 10.1038/ncomms6101 25290058 PMC4205890

[B46] Van Doorn R. Van Horssen J. Verzijl D. Witte M. Ronken E. Van Het Hof B. (2010). Sphingosine 1-phosphate receptor 1 and 3 are upregulated in multiple sclerosis lesions. *Glia* 58 1465–1476. 10.1002/glia.21021 20648639

[B47] Williams A. Piaton G. Lubetzki C. (2007). Astrocytes–friends or foes in multiple sclerosis? *Glia* 55 1300–1312. 10.1002/glia.20546 17626262

[B48] Yamada M. Mizuguchi M. Otsuka N. Ikeda K. Takahashi H. (1997). Ultrastructural localization of CD38 immunoreactivity in rat brain. *Brain Res.* 756 52–60. 10.1016/s0006-8993(97)00117-0 9187313

