# Supplementary figures and images for: Deletion of CD38 Suppresses Glial Activation and Neuroinflammation in a Mouse Model of Demyelination
Source: Front Cell Neurosci. 2019 Jun 6;13:258. doi: 10.3389/fncel.2019.00258 (PMC6563778; doi:10.3389/fncel.2019.00258)

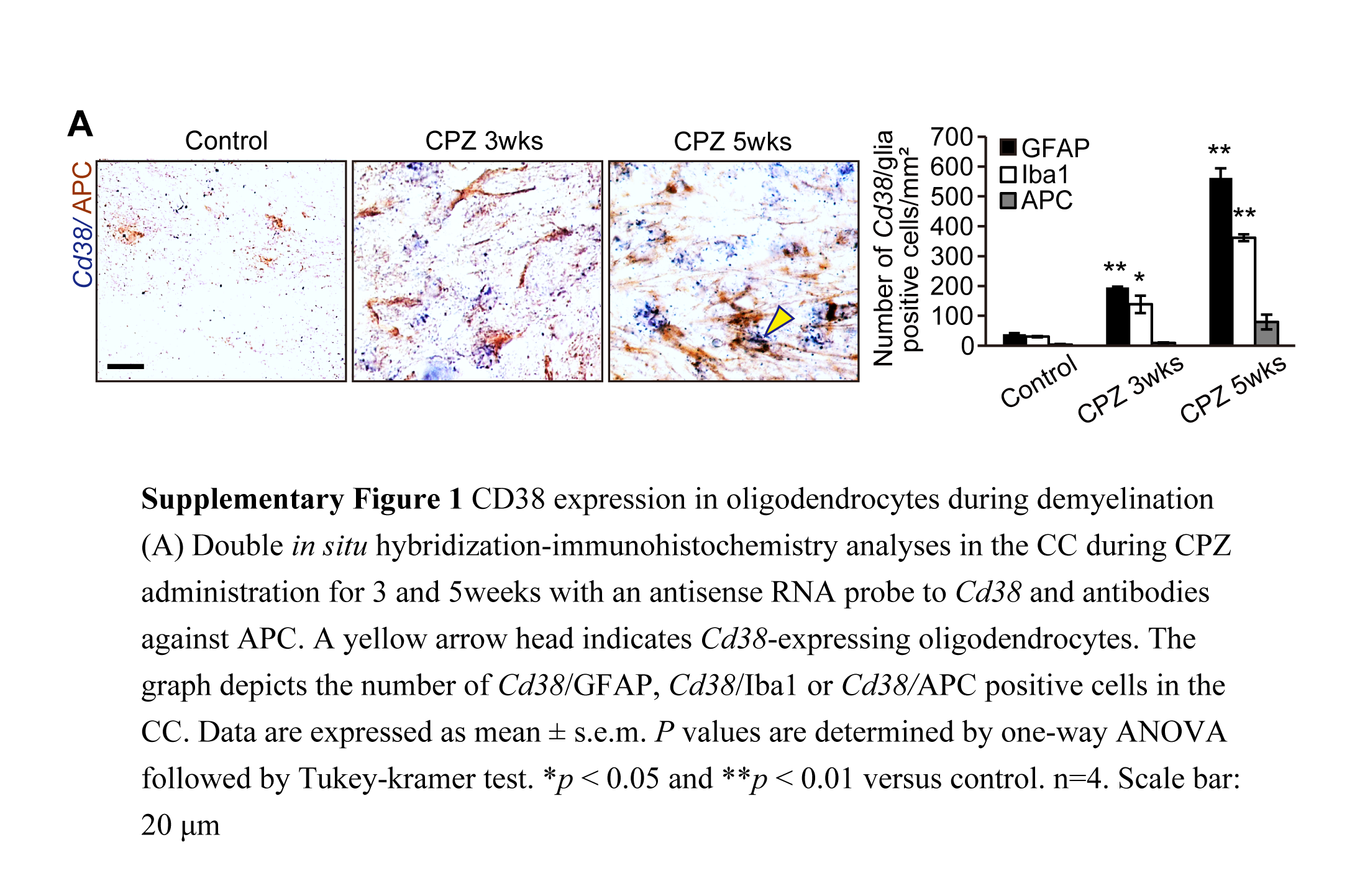

Supplement: Supplementary file 1 [file Image_1.TIF]

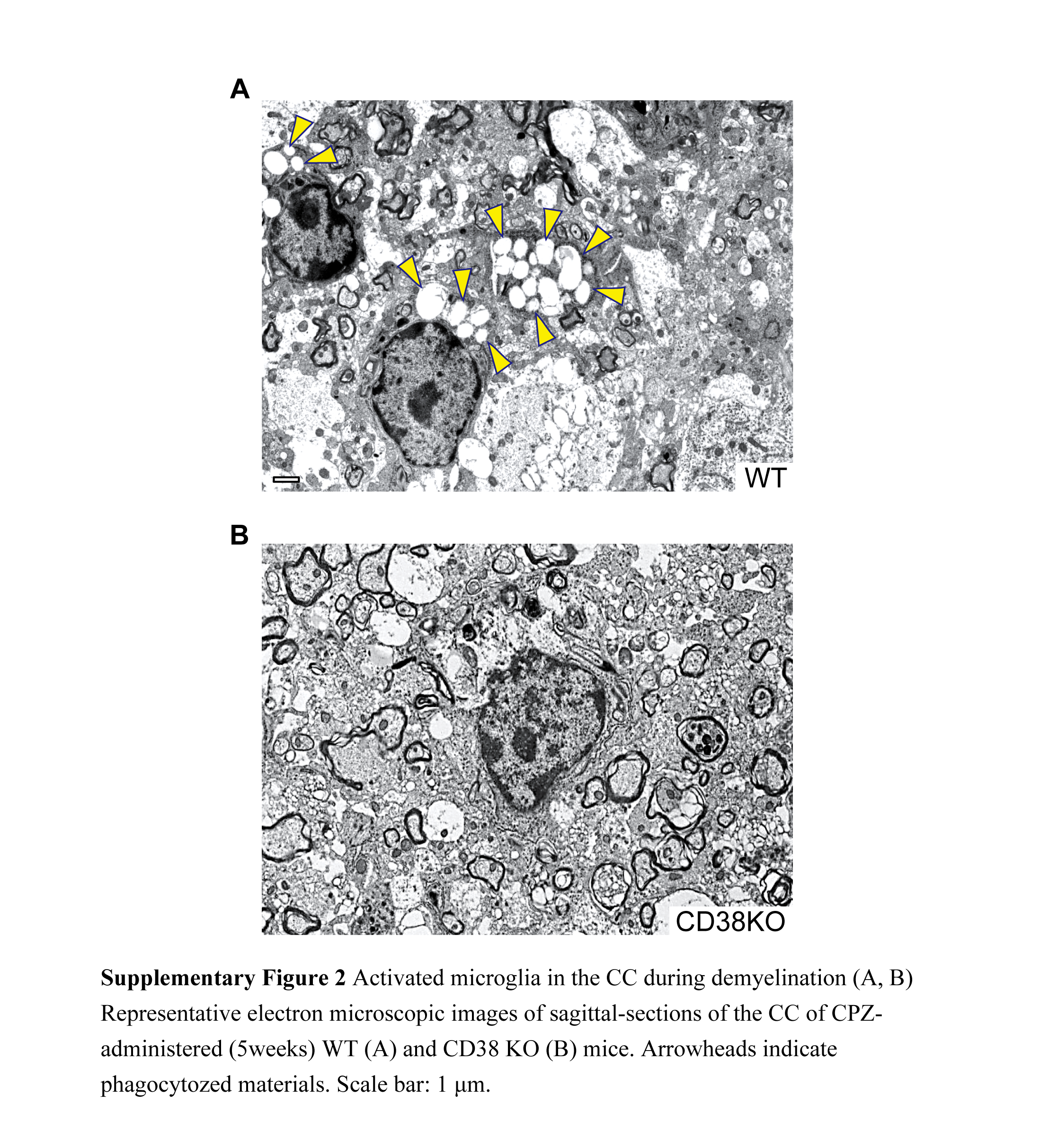

Supplement: Supplementary file 2 [file Image_2.TIF]

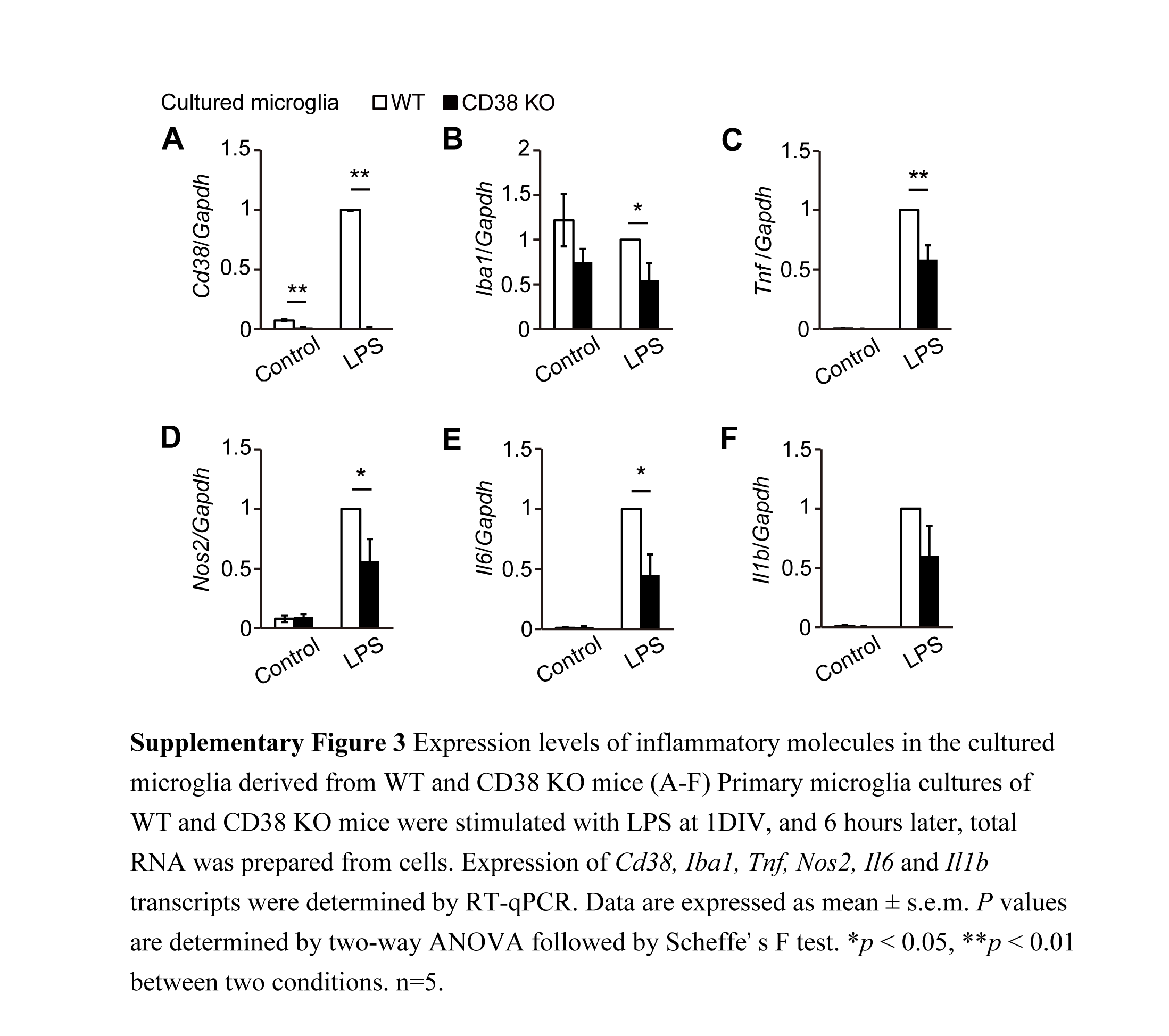

Supplement: Supplementary file 3 [file Image_3.TIF]
