# Supplementary material for: Deletion of CD38 Suppresses Glial Activation and Neuroinflammation in a Mouse Model of Demyelination
Source: Front Cell Neurosci. 2019 Jun 6;13:258. doi: 10.3389/fncel.2019.00258 (PMC6563778; doi:10.3389/fncel.2019.00258)
Supplement: Supplementary file 4 [file Table_1.DOCX]

**Supplemental Table 1 Primer sequences used for qRT-PCR analysis**

| Genes | Species | Forward | Reverse |
| --- | --- | --- | --- |
| *Cd38*  *Gfap*  *Iba1*  *Tnf*  *Il1b*  *Il6*  *Nos2*  *Ccl2*  *Ccl3*  *Cxcl10*  *Cxcl12*  *Cxcr4*  *Cd68*  *Trem2*  *Gapdh* | Mouse  Mouse  Mouse  Mouse  Mouse  Mouse  Mouse  Mouse  Mouse  Mouse  Mouse  Mouse  Mouse  Mouse  Mouse | CGAAGGAGCTTCCAGTAACG  CCCTGGCTCGTGTGGATTT  CAGCAATGATGAGGATCTGC  CAAGCCTGTAGCCCACGTCG  AAGCTCTCCACCTCAATGGA  CCGGAGAGGAGACTTCACAG  CAATGGTTGGTACATGGGCAC  CCAGCAAGATGATCCCAATG  ACCATGACACTCTGCAACCA  GCTGCAACTGCATCCATATC  TGCATCAGTGACGGTAAACCA  ACGGCTGTAGAGCGAGTGTT  CCACAGGCAGCACAGTGGACA  CTCCAGGAATCAAGAGACCTCC  ACCCAGAAGACTGTGGATGG | TGGCAGGCCTGTAGTTATCC  GACCGATACCACTCCTCTGTC  CCAAGTTTCTCCAGCATTCG  GATTGACCTCAGCGCTGAGT  AGGTGCTGATGTACCAGTTG  TCCACGATTTCCCAGAGAAC  GAGGGACCAGCCAAATCCAGT  TCTGGACCCATTCCTTCTTG  GTGGAATCTTCCGGCTGTAG  GTGGCAATGATCTCAACACG  TTCTTCAGCCGTGCAACAATC  CCGTCATGCTCCTTAGCTTC  TCCACAGCAGAAGCTTTGGCCC  CCGGGTCCAGTGAGGATCT  CACATTGGGGGTAGGAACAC |
