# Supplementary material for: Deletion of CD38 Suppresses Glial Activation and Neuroinflammation in a Mouse Model of Demyelination
Source: Front Cell Neurosci. 2019 Jun 6;13:258. doi: 10.3389/fncel.2019.00258 (PMC6563778; doi:10.3389/fncel.2019.00258)
Supplement: Supplementary file 5 [file Table_2.DOCX]

Supplementary table 2

| Antibody Name | Vender, Catalog number and Antibody ID according to Research Resource Identifies (RRIDs) | Dilution |
| --- | --- | --- |
| **Antibody used for Western Blotting** | | |
| Sheep anti-CD38 | R and D Systems Cat# AF4947, RRID:AB_1241945 | 1:500 |
| Rabbit anti-glial fibrillary acidic protein (GFAP) | Sigma-Aldrich Cat# G9269, RRID:AB_477035 | 1:5000 |
| Rabbit anti-ionized calcium binding adaptor molecule 1 (Iba1) | Wako Cat# 019-19741,  RRID:AB_839504 | 1:500 |
| Mouse anti-GAPDH | Wako Cat# 015-25473,  RRID:AB_2665526 | 1:2000 |
| Goat anti-rabbit | Santa Cruz Biotechnology Cat# sc-2004, RRID:AB_631746 | 1:5000 |
| Mouse anti-goat | Santa Cruz Biotechnology Cat# sc-2354, RRID:AB_628490 | 1:1000 |
| m-IgGκ BP-HRP Antibody | Santa Cruz Biotechnology Cat# sc-516102, RRID:AB_2687626 | 1:5000 |
| **Antibody used for Immunohistochemistry/ in situ hybridization** | | |
| Rat anti- myelin basic protein (MBP) | Millipore Cat# MAB386,  RRID:AB_94975 | 1:100 |
| Mouse anti- Amyloid Precursor Protein (APP) | Millipore Cat# MAB348,  RRID:AB_94882 | 1:200 |
| Rabbit anti-GFAP | Sigma-Aldrich Cat# G9269, RRID:AB_477035 | 1:200 |
| Rabbit anti-Iba1 | Wako Cat# 019-19741,  RRID:AB_839504 | 1:200 |
| Mouse anti-adenomatous polyposis coli (APC) | Millipore Cat# OP80,  RRID:AB_2057371 | 1:200 |
| Goat anti-Rat | Vector Laboratories Cat# MP-7444, RRID:AB_2336530 |  |
| Horse anti-mouse | Vector Laboratories Cat# MP-7402, RRID:AB_2336528 |  |
| Horse anti-rabbit | Vector Laboratories Cat# MP-7401, RRID:AB_2336529 |  |
| Antibody Name | Vender, Catalog number and Antibody ID according to Research Resource Identifies (RRIDs) | Dilution |
| **Antibody used for Immunofluorescence** | | |
| Mouse anti-APC | Calbiochem, OP80 | 1:100 |
| Mouse anti-Neurofilament H, nonphosphorylated (SMI-32) | BioLegend Cat# 801701  AB_2564642 | 1:500 |
| Rabbit anti-GFAP | Sigma-Aldrich Cat# G9269, RRID:AB_477035 | 1:500 |
| Rabbit anti-Iba1 | Wako Cat# 019-19741,  RRID:AB_839504 | 1:500 |
| (4’6-diamidino-2-phenylindole,  dihydrochloride)DAPI | Thermo Fisher Scientific Cat# D1306, RRID:AB_2629482 | 1:5000 |
| Donkey anti-mouse,  Alexa Fluor 488 -conjugated | Thermo Fisher Scientific Cat# A-21202, RRID:AB_141607 | 1:200 |
| Donkey anti-rabbit,  Alexa Fluor 488 -conjugated | Thermo Fisher Scientific Cat# A-21206, RRID:AB_2535792 | 1:200 |
| Goat anti-mouse, Cy3-conjugated | Millipore Cat# AP124C,  RRID:AB_92459 | 1:200 |
| Goat anti-rabbit, Cy3-conjugated | Millipore Cat# AP132C,  RRID:AB_92489 | 1:200 |
| **Antibody used for glial cell culture** | | |
| CD11b (microglia) microbeads conjugated to monoclonal anti-human/mouse CD11b antibody | Miltenyi Biotec, 130-093-634 |  |
